# Self-organization and culture of Mesenchymal Stem Cell spheroids in acoustic levitation

**DOI:** 10.1038/s41598-021-87459-6

**Published:** 2021-04-16

**Authors:** Nathan Jeger-Madiot, Lousineh Arakelian, Niclas Setterblad, Patrick Bruneval, Mauricio Hoyos, Jérôme Larghero, Jean-Luc Aider

**Affiliations:** 1 Laboratoire de Physique et Mécanique des Milieux Hétérogènes (PMMH), UMR 7636 CNRS, ESPCI Paris, PSL, Paris Sciences et Lettres University, Sorbonne Université, Université de Paris 1, Paris, 75005 France; 2 Unité de Thérapie Cellulaire, APHP, Hôpital Saint-Louis, 1 avenue Claude Vellefaux, 75010 Paris, France; 3 Université de Paris, Inserm U976 et CIC de Biothérapies CBT501, 75010 Paris, France; 4 Technological Core facility of the Institut de Recherche Saint-Louis, Université Paris-Diderot and Inserm, Hôpital Saint-Louis, Paris, France; 5 INSERM U970-PARCC, Paris, France

**Keywords:** Biomaterials, Stem-cell biotechnology, Tissue engineering, Biomedical engineering, Acoustics

## Abstract

In recent years, 3D cell culture models such as spheroid or organoid technologies have known important developments. Many studies have shown that 3D cultures exhibit better biomimetic properties compared to 2D cultures. These properties are important for in-vitro modeling systems, as well as for in-vivo cell therapies and tissue engineering approaches. A reliable use of 3D cellular models still requires standardized protocols with well-controlled and reproducible parameters. To address this challenge, a robust and scaffold-free approach is proposed, which relies on multi-trap acoustic levitation. This technology is successfully applied to Mesenchymal Stem Cells (MSCs) maintained in acoustic levitation over a 24-h period. During the culture, MSCs spontaneously self-organized from cell sheets to cell spheroids with a characteristic time of about 10 h. Each acoustofluidic chip could contain up to 30 spheroids in acoustic levitation and four chips could be ran in parallel, leading to the production of 120 spheroids per experiment. Various biological characterizations showed that the cells inside the spheroids were viable, maintained the expression of their cell surface markers and had a higher differentiation capacity compared to standard 2D culture conditions. These results open the path to long-time cell culture in acoustic levitation of cell sheets or spheroids for any type of cells.

## Introduction

3D cell cultures have shown closer biological properties to cells in a physiological context than 2D cell cultures^1,2^. Therefore they have become one of the main research and development topics in cell biology of recent years^3^. Nevertheless, the clinical translation of these models has been very limited due to a lack of reproducibility of the results^4^. Even though some spheroids have found their way to clinical trials, especially for applications such as bone regeneration, it has been more controversial for softer tissues^5^. Most protocols rely on the use of poorly defined hydrogels which affect cells with their chemical and biomechanical properties^6^. Furthermore, the use of recombinant growth factors or products from animal origins in these hydrogels is a major issue for human clinical application of these spheroids.

Alternatively, several scaffold-free methods, such as hanging drop^7^, non-adhesive surface^8^ or rotating bioreactor^9^, have shown spheroid fabrication as 3D cell culture models. Some disadvantages such as variation in spheroid size and shape still remain unsolved^10^. However, several methods create homogeneous spheroids, including liquid overlay technique, agarose mold wells and ultralow attachment spheroid microplates. Spheroid formation by these methods depends on the contractile properties of the cells and may be less convenient for low contractile cells^11^.

Thanks to its excellent biocompatibility and versatility, acoustic approaches are promising tools for contactless manipulation of cells^12^. Besides the various applications of cell focusing or separation^13^, acoustic methods have been used for tissue engineering and in vitro techniques^14^. Many works^15,16^ studied cell behavior with a short-time ultrasound exposure, classically around 1 h and used it to instantaneously shape complex geometries like layer^17^, multi-layer^18^ or spheroid^19^ in order to culture it in usual way.

Here, we report the fabrication and the culture of several spheroids over a 24-h period in an acoustofluidic chip with a well-controlled cell culture environment. The acoustic forces act on the suspended cells^20^ by clustering and trapping them in multiple disk-like layers in acoustic levitation, located at the different pressure nodes of a resonant cylindrical cavity^21^. After a self-organization step, the spheroids could be maintained and cultured in levitation, with a system of perfused culture medium.

We designed an acoustofluidic chip made of a PDMS (Polydimethylsiloxane) body bonded to a microscope cover-glass. We chose PDMS material because it is highly biocompatible and permeable to the air, making it ideal for cell culture^22^. It is also transparent, allowing optical accessibility and is easy to process.

**Figure 1:**
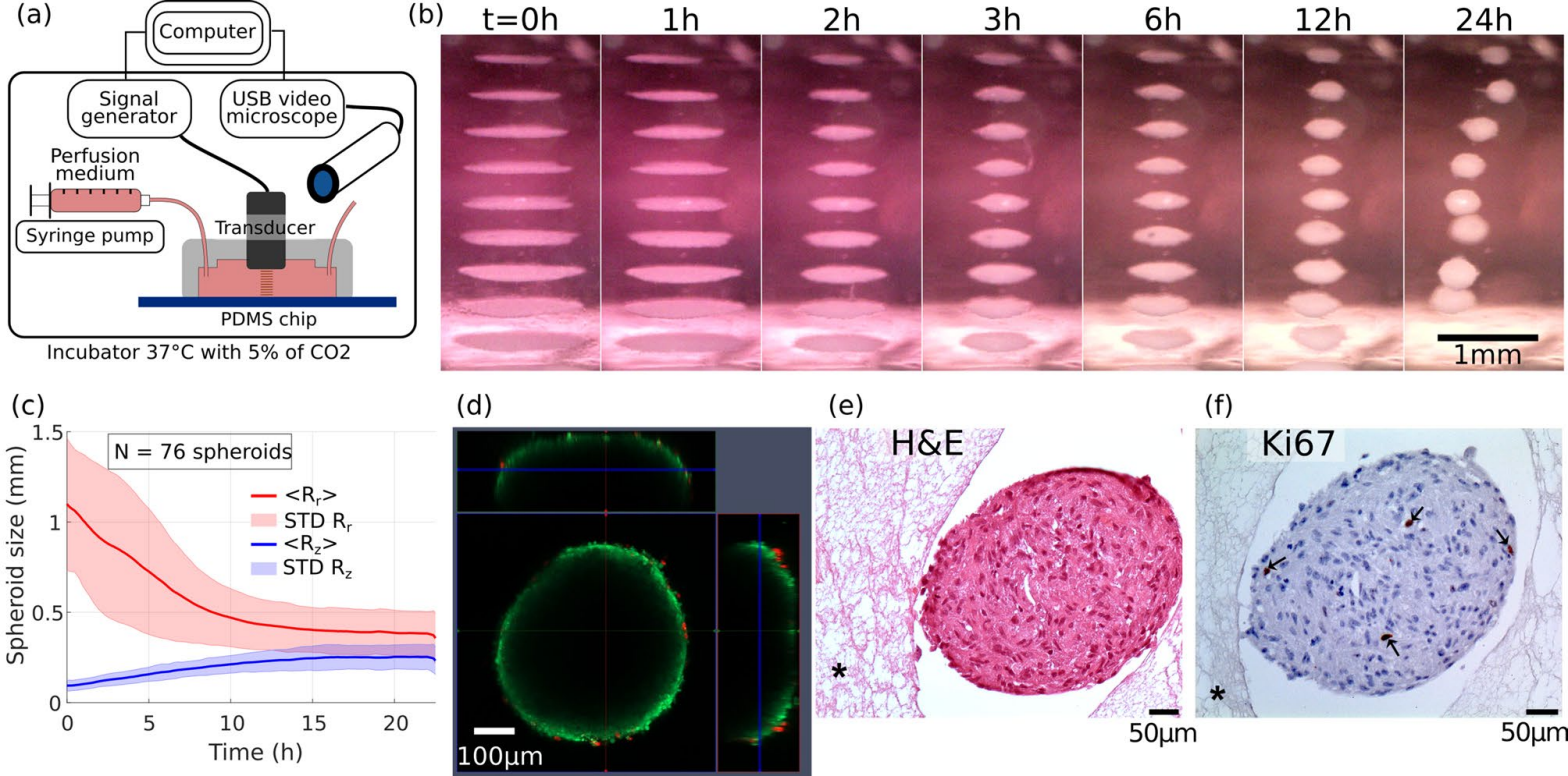
Self-organization of MSCs cells sheets into spheroid shapes and biological viability. (**a**) Presentation of the experimental setup. A computer drives the wave generation and the acquisition of images. The transducer converts the electrical energy into acoustic energy inside the chip cavity and produced the acoustic levitation of cells over multiple acoustic pressure nodes. A USB digital microscope took side-view pictures and time-lapses of the cell sheets in levitation and self-organization into spheroids. A syringe pump allows a continuous perfusion of the cell medium during the 24 h acoustic manipulation. The whole setup, apart from the computer and the generator, was put in an incubator for a well-controlled cell culture environment. (**b**) Time lapse snapshots of the cell self-organization. The whole time-lapse is available on the Supplementary Video 2. At the beginning, the cells were trapped in monolayers. Then, the cells sheets contracted and the cells self-organized to reach a stable spheroid shape. (**c**) Time-evolution of the axial $R_z$ and the radial $R_r$ dimensions study of the cells aggregates, averaged over 76 individuals, computed from the time-lapse observations. At $t=0\,{\mathrm {h}}$, the widths $R_r$ ranged from 0.75 to 1.5 mm depending on the aggregate location. However, all the heights were similar because of the monolayer shape. After 15 h of levitation, every MSC aggregates have reached a stable spheroid shape ($R_z \approx R_r$). (**d**) Confocal and fluorescence imagery of a spheroid just after the acoustic levitation. A live/dead kit was used to label the cells. Because of the limited optical penetration or the substrate diffusion, only the cells at the surface were visible and a majority of them showed a living signal. (**e**) Histology of MSC spheroids. After a 24 h levitation, spheroids were collected from the chip and were immediately fixed in parafolmaldehyde 4% overnight. The spheroids were then embedded in fibrin network (described by the symbol * on the images) for an easy handling of paraffin inclusion and histological preparation. Spheroids were stained with H&E for general structure evaluation and (**f**) Ki67 for proliferation.

A 2 MHz ultrasonic transducer (Fig. 1a) was inserted into the PDMS body, in direct contact with the fluid, closing the cylindrical cavity. The microscope cover-glass, facing the transducer, acts as an efficient acoustic reflector and allows an optical access to visualize the cell clusters. The geometry of our chip has been optimized in order to increase the number of acoustic traps per chip, up to 30, and to be easily parallelizable. In this study we have used four chips in parallel, leading to the culture of 120 cell spheroids per experiment. To maintain sterility, the chips are single use and disposable.

We first demonstrated the functionality of our system using a suspension of $10\,{\upmu }{\mathrm {m}}$ polystyrene beads close to the targeted cells dimensions. As soon as the acoustic generator was turned on, the particles were trapped in the acoustic nodes as monolayers and remained stable as long as needed. This result is illustrated for a 90 h experiment on the Supplementary Video 1.

We then evaluated our system for culturing human cells. MSCs are primary cells that can been isolated from various tissue sources including the umbilical cords, adipose tissue and the bone marrow. They are characterized by their ability to adhere to plastic, by the expression of certain surface markers, as well as their capacity to differentiate into adipocytes, osteoblasts and chondrocytes. These cells are used in many clinical and tissue engineering applications. We decided to use this cell model as a proof of concept for our system. We isolated these cells from human adipose tissue.

## Results

### 24 hours acoustic levitation of MSCs and self-organization into spheroids

For the acoustic levitation experiments, the cells were injected in the cavity at a concentration of 1.5 million cells/mL and cultured for 24 h. The setup has been optimized to allow a constant flow of nutrients. The cell culture medium was injected at a rate of $20\,{\upmu }{\mathrm {L}}/{\mathrm {h}}$, which allowed constant medium renewal without disturbing the cells aggregates in acoustic levitation. The observation of the cells’ collective behavior in levitation showed that they first formed monolayers in the same way as for the beads. Then, gradually, during the first few hours, they spontaneously reorganized and formed spheroids of a typical diameter close to $400\;\upmu {\mathrm {m}} \pm 60\;\upmu {\mathrm {m}}$ (Fig. 1b, Supplementary Video 2 and Supplementary Video 3 in the supplementary information). This behavior was simultaneously observed in all the cell layers in acoustic levitation. This illustrated the self-organizational behavior of living cells, which was not observed with polystyrene beads.

### Time evolution of the spheroid sizes

The dynamics of the self-organization into spheroids was studied through image processing of the video time series. The axial $R_z$ and radial $R_r$ dimensions of the spheroids were measured for every spheroid and averaged at each time step. We show on Fig. 1c the time evolution of the mean values $<R_z>(t)$ and $<R_r>(t)$. One can see that after 12 h, all the cell sheets have turned into stable spheroids elongated along the radial dimension. The average diameter of the spheroids, obtained on 76 samples, is $400\;\upmu {\mathrm {m}} \pm 60\;\upmu {\mathrm {m}}$.

### Viability and proliferation of the MSCs spheroids after the 24 hour acoustic levitation

We evaluated the viability of the cells after a 24 h culture in acoustic levitation using calcein and propidium iodide, which showed that the majority of the cells were alive at the surface of the spheroid (Fig. 1d). However, due to the optical limitation of the confocal microscopy or substrate diffusion, it was not possible to visualize the core of the spheroids with this technique. We therefore evaluated the integrity of the structures by histology. Hematoxylin and eosin (H&E) staining showed that the spheroids were filled structures, with no necrosis in their cores (Fig. 1e). Furthermore, the intact form of the cell nuclei indicated that they were not apoptotic. The evaluation of cell proliferation by Ki67 staining showed that most cells were not proliferative but a few cells still stained positive, despite being in levitation (Fig. 1f). These results indicate a behavior of the cells inside the spheroids close to that of cells within tissues, which are mainly quiescent while only a few cells proliferate^23^.

### Differentiation of the MSCs spheroids

**Figure 2:**
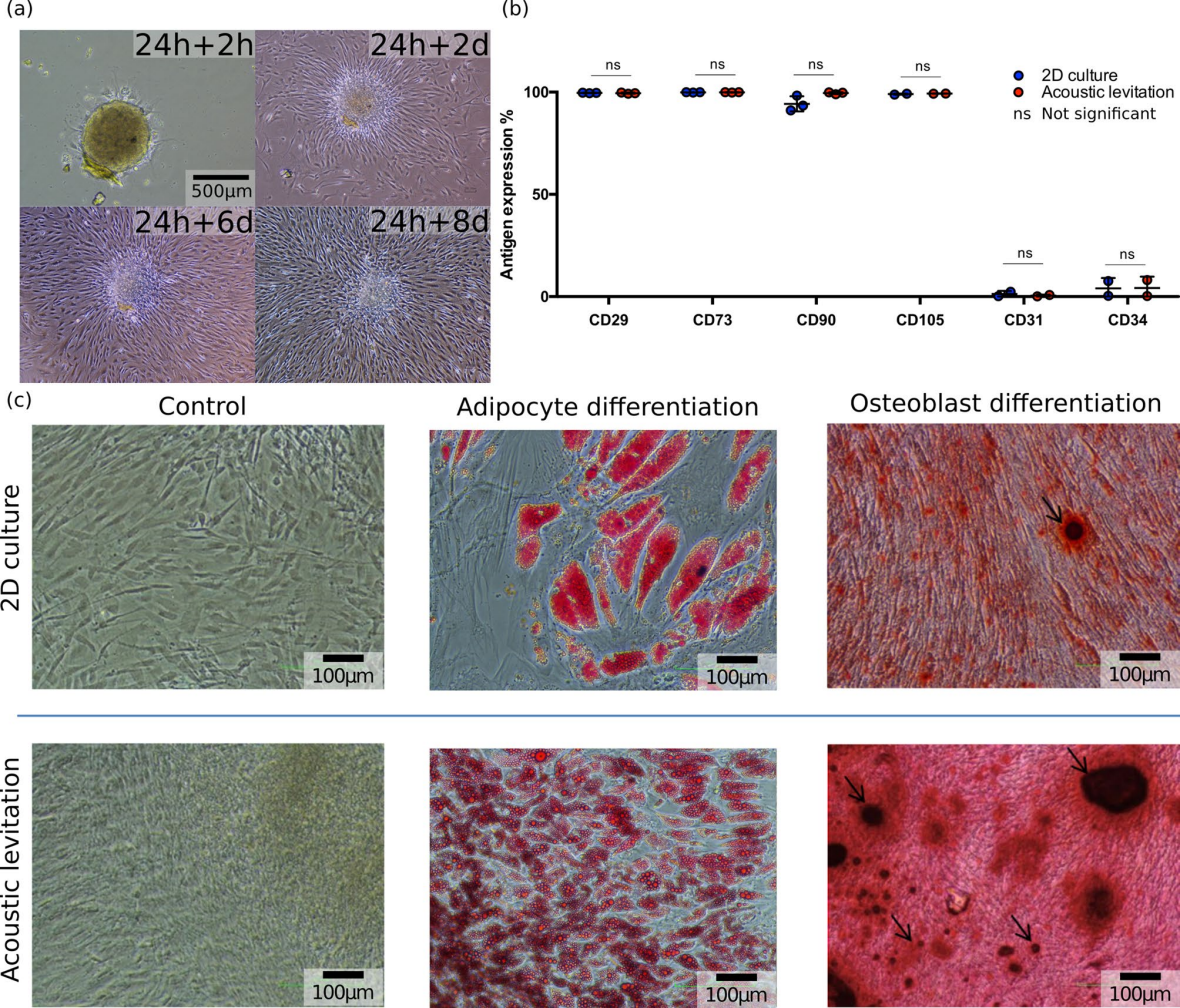
Biological evaluation of MSCs in levitation. (**a**) Pictures of a spheroid recovered after the 24 h levitation and reseeded in a classical culture plate. 2 h after the levitation, the spheroid began to adhere to the plastic surface. Then, during the 8 following days, the cells spread out onto the plate. (**b**) Evaluation of MSC cell surface markers by flow cytometry. After a 24 h levitation, the spheroids were expanded in polystyrene dishes. A week later, cells were detached, stained and analyzed for the expression of classic MSC markers by flow cytometry. Here the results are expressed as the percentage of positive cells. Statistical tests were performed with a t-test. The p-values are not significant (${\text {p-values}} > 0.05$), see Supplementary Table S1. (**c**) Differentiation of MSCs into adipocytes and osteoblasts. After a 24 h acoustic levitation, spheroids were reseeded in 24 well plates. Once they adhered, they were treated with MSC expansion medium (control), adipocyte or osteoblast differentiation media. A 2D confluent culture of cells, which did not undergo acoustic levitation was used as a control condition. Adipocyte differentiation was revealed by the staining of lipid vesicles by oil-red O and osteoblasts were revealed by alizarin-red staining.

The spheroids were then reseeded in culture plates (Fig. 2a), and their attachment and spreading were observed with a video microscope (Nikon Biostation, Supplementary Video 7 and Supplementary Video 4 in the supplementary information). The spheroids maintained their potential to re-adhere to plastic and MSCs were shown to proliferate and spread out. In these conditions, MSCs expressed the standard surface markers of mesenchymal cells, thus showing that levitation did not alter these properties (Fig. 2b). Furthermore, differentiation into adipocytes after attachment to plastic showed that the cells closest to the center of the spheroid had the highest potential of differentiation and lipid accumulation (Fig. 2c). The number of adipocytes observed with the spheroid was much higher than the control condition in 2D culture. When differentiated along the osteoblastic lineage, MSCs cultured in levitation showed higher number and a larger size of osteoblast nodules than in 2D control conditions (Fig. 2c), indicating that the physical effects induced by levitation increased the potential of osteoblastic differentiation.

## Discussion

We hereby demonstrate the development of a robust and reproducible cell culture system based on acoustic levitation using a PDMS chip and an ultrasound transducer, with the possibility of a constant flow, which allows culture medium renewal. Even though previous studies have shown the possibility of using acoustic levitation for cell sheet and aggregate formation, these studies were limited to a very short treatment period usually less than 1 h^12^. Only a few studies pushed the culture time beyond 24 h. However, either the device was limited to a single aggregate^24^ or the method produced small aggregates with no levitation ($<150\;\upmu {\mathrm {m}}$)^25^. Thanks to its versatility and ease of implementation, our cell culture in multi-trap acoustic levitation (CCMAL) device allows the production and the culture of hundreds of large spheroids with a reproducible size. The CCMAL setup can be easily scaled up to culture thousands of spheroids in acoustic levitation in parallel. It is well adapted for MSC cultures, preserving their viability, proliferation and characteristic surface markers. It also increases their potential of differentiation. It has been previously shown that high density of MSCs in 2D culture, as well as spheroid formation in low attachment dishes^26^, or the application of ultrasound^27^ enhances their osteoblastic potential. In our system, the rapid formation of spheroids mimics both high confluency of 2D cultures and spheroid formation owing to the acoustic confinement (aggregation induced by the axial and transverse components of the ARF). Unlike spheroid formation methods which rely only on the contractile properties of cells, our system has the advantage of trapping cells in acoustic traps and forcing them to interact which facilitates cell sheet and spheroid formation. This approach opens the path to a better understanding of these phenomena and application to many other different types of cells. It also demonstrates the use of acoustic levitation as a new method of long-term, reproducible cell culture process for scaffold-free tissue engineering purposes such as organoid formation and multicellular co-cultures.

## Methods

### Acoustofluidic setup

For each experiment, we ran four cell culture in multi-trap acoustic levitation (CCMAL) devices in parallel in order to increase the number of spheroids and maximize the cell preparation. One has to control the acoustic levitation process, the optical observation, the fluid perfusion as well as the biological part from the cell preparation to the post-levitation analysis and the collect of all samples.

The ultrasonic waves were generated by a transducer driven by an arbitrary waveform generator (Handyscope HS5 from TiePie Engineering, Sneek, Netherlands) monitored by a computer. Each output of the signal generators supplied two ultrasonic transducers (2 MHz SignalProcessing, Savigny, Switzerland) with a sinusoidal waveform of amplitude $7{\mathrm {V}}_{\mathrm {pp}}$ and frequency 2.15 MHz. The wave parameters were chosen to optimize the levitation process while avoiding undesired phenomena like acoustic streaming.

The chip is composed by a PDMS (Polydimethylsiloxane, RTV 615, Neyco, Vanves) body bonded on microscope cover-glass and a mono-element ultrasonic transducer. The geometry of the PDMS chip is designed to allow the insertion of the ultrasonic transducer inside the PDMS body. The transducer closed the acoustofluidic cavity. One side of the resonant cavity is ultrasound emitter, in contact with the fluid, avoiding acoustic losses through various interfaces, while on the other side the microscope cover-glass acts as a powerful acoustic reflector and allows an optical access. The overall acoustic resonant cavity is simple and efficient.

To shape the PDMS body, we use a negative mold made with a 3D printer (3D Form 2 from Formlabs Inc., Somerville, United States). Then the PDMS is poured in and put in the oven at $70\,^\circ {\mathrm {C}}$ for 24 h. After unmolding the solid PDMS body is bounded on a cover-glass with a plasma cleaner.

**Figure 3:**
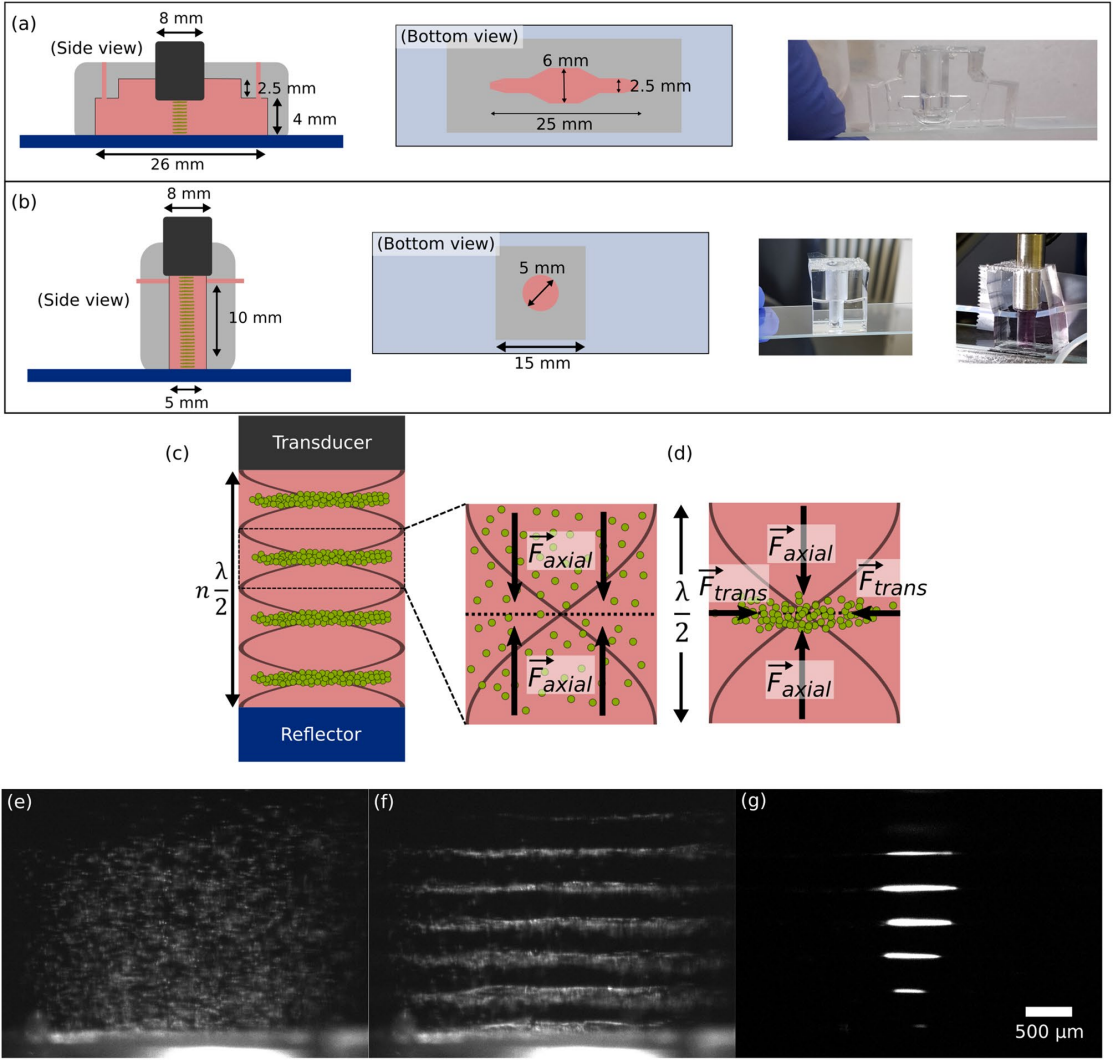
(**a**) Chip geometry 1 (**b**) Chip geometry 2 (**c**) Schema of the multilayer acoustic cavity (**d**) The suspension of cells particles undergoes the Acoustic Radiation Force (ARF). The cells are first focused in the nodal plane and then, trapped by the transverse component of the ARF. The cavity dimensions are adjusted to have multiple clusters. Example of the levitation and aggregation process: (**e**) Suspended particles are injected in the cavity. Here is $15\,\upmu {\mathrm {m}}$ diameter polystyrene beads. (**f**) Axial acoustic radiation forces are dominating and bring the particles in the pressure nodal plane. (**g**) Therefore, the transverse forces trap particles in a circular monolayer.

Because of this fabrication process, the geometry of the chip is highly versatile. We settled on two geometries. The first chip (see Fig. 3a) includes the levitation cavity with 11 nodes, a channel and a simple bubble trap. Our manufacture process makes the sidewall perfectly transparent to visible optical waves and allows the observation from the side of the acoustic levitation process over the entire cavity. To increase the spheroid number from 11 up to 27 we designed a second chip considering the acoustic parameters (See Supplementary Fig. S 1 and Supplementary Video 6). The geometry (see Fig. 3b) consists in a cylindrical well of diameter $D = 5\,{\mathrm {mm}}$ and height $10\,{\mathrm {mm}}$. The optical access is still possible but with a lower quality. To monitor the formation of the cells sheets and the spheroids self-organization from the side view, we used four usb video microscopes (Edge and AM 2111 from Dinolite company, Taipei, Taiwan).

The culture medium was continuously perfused in the chip with a syringe pump (Pump 11 Elite from Harvard Apparatus, Holliston, Unisted States) in a withdrawal mode with a flowrate of $20\,\upmu {\mathrm {L}}/{\mathrm {h}}$ small enough to avoid any perturbations inside the acoustofluidic cavity.

### Image processing for the study of the spheroid size

In order to characterize the time evolution of the sizes of the spheroids, we developed a Matlab image analysis code. It works automatically with a few user-defined parameters. In particular, we manually defined the regions of interest (ROIs) for each spheroid. Then an ellipsoid is fitted on the binarized image. The section of the original image passing through the ellipse center is selected in the axial and radial directions. We stored the width of the 70% thresholded curves of the X and Y sections (See Supplementary Video 5). For the whole image sequence, ROIs were defined automatically from the ellipsoid characteristics of the previous image. We obtained the time evolution of the axial and radial dimensions of the spheroid with a 1-min time step. We finally applied a moving average of 30 points to smooth the signal fluctuations. Afterwards, the average and the standard-deviation were computed.

### MSC isolation and characterization

MSCs were isolated from adipose tissue of the thigh of a healthy donor, after a signed consent, according to the French regulations. MSCs were obtained after the digestion of adipose tissue with collagenase NB6 (Serva electrophoresis, Heidelberg, Germany). The adipose tissue was placed in 40 mL of collagenase NB6 diluted in $\alpha$-MEM medium at a final concentration of $5\,\upmu {\mathrm {g}}/{\mathrm {mL}}$ in a 50 mL conical tube (Falcon, Dutscher, Bernolsheim, France). After a 2 h incubation at $37\,^{\circ }{\mathrm {C}}$, the digested tissue was filtered through a cell strain with pores of $100\,\upmu {\mathrm {m}}$ to remove the remaining tissue. The strained cells were then centrifuged and plated in a cell factory (Thermofisher, Nunc, Waltham, United States) for expanding in MSC culture medium composed of $\alpha$-MEM (Gibco, Thermofisher, Waltham, United States) supplemented with 10% fetal bovine serum (FBS, Biowest, Nuaillé, France) and 1% antibiotic/antimycotic mix (Anti-Anti 100×, Gibco, Thermofisher, Waltham, United States).

At the second passage, cells were characterized by flow cytometry using a panel of MSC positive markers including CD29-PE (MACS Miltenyi, Bergish Gladbach, Germany), CD73-PE (cat number: 550257, bd, New Jersey, United States), CD90-FITC (cat number: 555595, bd), CD105-PE (cat number: 560839, bd), and negative markers including CD31-FITC (cat number: 555445, bd) and CD34-APC (cat number: 555824, bd). Unstained cells and cells stained with control isotypes have been used as negative control.

### MSC characterization after acoustic levitation

After 24 h of acoustic levitation, the spheroids were collected and immediately fixed overnight in paraformaldehyde 4% (Alfa Aesar, Kandel, Germany) diluted in PBS (Eurobio Scientific, Les Ulis, France). They were then embedded in a fibrin gel to facilitate manipulation for preparing samples. The fibrin gel containing the spheroids was included in paraffin and $5\,\upmu {\mathrm {m}}$ thick sections were cut. The slices were stained with H&E for general structure. Ki67 immunohistochemistry was performed with anti-ki67 antibody (Agilent-Dako, Santa Clara, United States) 3-step immunoperoxidase technique.

### MSC viability and adherence after acoustic levitation

To evaluate the capacity of MSC spheroids, which also shows their viability, they were placed in 24 well dishes and were observed during 24 h in an Incucyte system (Sartorius, Göttingen, Germany). Cell viability was further evaluated with a LIVE/DEAD Viability/Cytotoxicity Kit (Invitrogen, Carlsbad, United States). The components (calcein and propidium iodide) were diluted in PBS according to the provided instructions of the kit and the spheroids were incubated in the solution for 30 mins in an Ibidi dish prior to observation with a confocal microscope (Zeiss LSM 780 AiryScan, Oberkochen, Germany).

### Evaluation of MSC surface markers and potential of differentiation

After 24 h of acoustic levitation, MSC spheroids were collected and reseeded in $75\,{\mathrm {cm}}^2$ for expanding in MSC culture medium. About a week later, cells were detached and evaluated by flow cytometry using a panel of MSC positive and negative markers including CD31, CD34, CD29, CD73, CD90 and CD105 (see Supplementary Fig. S2 and Fig. S3). Data were acquired and analyzed with an Attune flow cytometer (Thermofisher, Nunc, Waltham, United States).

The potential of differentiation of the aggregates into adipocytes and osteoblasts was also evaluated. For this purpose, the spheroids were collected and reseeded in a 24 wells plate at a density of one spheroid/well in MSC expansion medium. A 2D culture of cells at the same passage that had not undergone levitation was also used as control. These cells were about $70\%$ confluent at the beginning of the differentiation.

After allowing the cells to adhere and expand for 3 days, the cell culture medium was replaced by adipocyte differentiation medium or osteoblast differentiation medium (StemXVivo, R&D systems Inc., Minneapolis, United States). MSC expansion medium was also used as the control condition. After a 2-weeks culture, cells were fixed with 4% paraformaldehyde. Adipocyte differentiation was permeabilized with isopropanol for 5 mins and the lipid vesicles were stained with oil red-O (Sigma-Aldrich, Munich, Germany). Osteoblasts were stained with alizarin red (Sigma-Aldrich).

### Ethical statement

We declare that all methods in the manuscript were carried out in accordance with relevant guidelines and regulations given by Nature Scientific Reports. All procedures involving patients were conducted according to the Helsinki Declaration. Mesenchymal Stem Cells were isolated from the adipose tissue of a male donor, obtained at Necker-Enfants Malades hospital in Paris, France. The adipose tissue was the surgical leftover and was used for research purposes after a signed consent from the donor and his parents as the legal tutors, according to the French bioethical and medical research regulations. In France, according to the law “loi Jardé” (article L. 1121-1 of the public health code) governing scientific research on human subjects and tissue samples, the surgical leftover can be used for scientific research without the prior approval of an ethical committee.

## Supplementary Information

Supplementary Information.

Supplementary Video 1

Supplementary Video 2

Supplementary Video 3

Supplementary Video 4

Supplementary Video 5

Supplementary Video 6

Supplementary Video 7
