# Author Correction: Self‑organization and culture of Mesenchymal Stem Cell spheroids in acoustic levitation

**DOI:** 10.1038/s41598-021-91887-9

**Published:** 2021-06-15

**Authors:** Nathan Jeger‑Madiot, Lousineh Arakelian, Niclas Setterblad, Patrick Bruneval, Mauricio Hoyos, Jérôme Larghero, Jean‑Luc Aider

**Affiliations:** 1 Laboratoire de Physique et Mécanique des Milieux Hétérogènes (PMMH), UMR 7636 CNRS, ESPCI Paris, PSL, Paris Sciences et Lettres University, Sorbonne Université, Université de Paris 1, 75005 Paris, France; 2 Unité de Thérapie Cellulaire, APHP, Hôpital Saint-Louis, 1 avenue Claude Vellefaux, 75010 Paris, France; 3 Université de Paris, Inserm U976 et CIC de Biothérapies CBT501, 75010 Paris, France; 4 Université Paris-Diderot and Inserm, Hôpital Saint-Louis, Paris, France; 5 INSERM U970-PARCC, Paris, France

Correction to: *Scientific Reports* 10.1038/s41598-021-87459-6, published online 16 April 2021

The original version of this Article contained errors, where references to the Supplementary Video files were not linked correctly.

Additionally, in the Supplementary Information file, Supplementary Figures S1-3 were given as “Figure 4”, “Figure 5” and “Figure 6” respectively, and Supplementary Table S1 was given as “Table 1”.

These errors have been corrected in the original version of the Article, and in the accompanying Supplementary Information file.

