# Supplementary material for: Self-organization and culture of Mesenchymal Stem Cell spheroids in acoustic levitation
Source: Sci Rep. 2021 Apr 16;11:8355. doi: 10.1038/s41598-021-87459-6 (PMC8052426; doi:10.1038/s41598-021-87459-6)
Supplement: Supplementary file 1 — Supplementary Information. [file 41598_2021_87459_MOESM1_ESM.pdf]

Self-organization and culture of Mesenchymal Stem Cell spheroids in acoustic levitation

Nathan Jeger-Madiot[1,*+], Lousineh Arakelian[2,3,*+], Niclas Setterblad[4], Patrick Bruneval[5], Mauricio Hoyos[1], Jérôme Larghero[2,3], and Jean-Luc Aider[1]

[1]Laboratoire de Physique et Mécanique des Milieux Hétérogènes (PMMH), UMR 7636 CNRS, ESPCI Paris, PSL, Paris Sciences et Lettres University, Sorbonne Université, Université de Paris 1, Paris, 75005, France
[2]Unité de Thérapie Cellulaire, APHP, Hôpital Saint-Louis, 1 avenue Claude Vellefaux, F-75010 Paris, France
[3]Université de Paris, Inserm U976 et CIC de Biothérapies CBT501, F-75010 Paris, France
[4]Technological Core facility of the Institut de Recherche Saint-Louis, Université Paris-Diderot and Inserm, Hôpital Saint-Louis, Paris, France
[5]INSERM U970-PARCC, Paris, France
[*]nathan.jeger-madiot@espci.fr; lousineh.arakelian@aphp.fr
[+]these authors contributed equally to this work

Supplementary informations

Acoustofluidic principles

The acoustic levitation describes the fact that an object is suspended in a medium by the action of the Acoustic Radiation Force (ARF) against the gravity. The ARF results from the time-averaged acoustic quantities from scattering of the acoustic waves on the objects. The ARF is explained by the second order perturbation theory of the Navier-stokes equation. The first analytical models of the ARF applied to particles date back in 1934 and make strong simplifying hypothesis like considering incompressible spheres[1]. Then, the compressibility was taken into account by the Yosioka model[2] but with sound field assumptions (considering a plane progressive or a standing-plane wave). Gor'kov[3] summarized and generalized the previous models with the following assumptions: the surrounding fluid is inviscid and the particles are much smaller than the acoustic wavelength λ.

In our experiments we used the axial and the transverse components of the ARF: the axial component moves the cells toward the pressure nodes (Fig. 3 c,e,f in the main article), while the transverse components forces the cells to gather at the location of maximum acoustic energy, roughly at the center of the cylindrical cavity (Fig. 3 d, g in the main article). The location and strength of the acoustic traps can be adjusted by changing the acoustic frequency f_{ac}, as shown by[4]. The acoustic levitation process is mainly due to the primary ARF given by the equation (1).

$$\vec{F}_{ac} = \frac{\pi}{4} < E_{ac} > k d_p^3 F_Y \sin(2kz)\vec{e}_z \tag{1}$$

with $< E_{ac} >$ for time-average of the acoustic energy density, $k = \frac{2\pi}{\lambda}$ the wave number of the acoustic plane wave with frequency f, c_m the acoustic celerity in the medium, d_p the particle or cell diameter, and F_Y the contrast factor. The z variable represents the axial position. The cells are affected by the primary ARF because of their acoustic contrast with the suspending medium quantified by the acoustic contrast factor F_Y :

$$F_Y = \frac{1 + \frac{2}{3}(1 - \frac{\rho_m}{\rho_c})}{2 + \frac{\rho_m}{\rho_c}} - \frac{\rho_m c_f^2}{3\rho_c c_c^2} \tag{2}$$

with c_p the acoustic celerity in the cell material, c_m the acoustic celerity in the medium, ρ_c the cell density and ρ_m the medium density.

The amplitude of the primary ARF typically ranges between 10^{-14} and 10^{-12} N (here for a $5V_{pp}$ input signal, the force is about 5×10^{-11} N).

In general, for cells and micro-organisms like bacteria, the contrast factor is positive thus they move toward the pressure nodes. The closer the cells go near the theoretical node, and the weaker the axial primary ARF will be until cancelling itself

when reaching the pressure node. From this observation, the transverse ARF becomes significant and is responsible of the cell aggregation in the nodal plane. For a radially symmetric acoustic wave, the transverse ARF can be described in the nodal plane with the equation equation (3)

$$F_T = d_p^3 \frac{3(\rho_c - \rho_m)}{\rho_m + 2\rho_c} \nabla < E_{ac} > \tag{3}$$

In this work we use a cylindrical transducer. The radial acoustic energy gradient is directed toward the axis center, where the acoustic energy is the highest. This region can be considered as an acoustic trap where any cells with a positive contrast factor, as well as most living micro-organisms, even motile like bacteria[5], can be trapped and contained within the acoustic force field[6].

A supplementary short-range force, called Bjerkness or Secondary Radiation Force (SRF), attracts cells close to each other until clustering. This is an inter-cells force due to the scattered acoustic field by the other cells. When cells are lined up perpendicular to the wave propagation (located in the nodal plane for instance) the force is attractive and can be expressed by equation (4)

$$F_r = \frac{\pi(\rho_c - \rho_m)^2 v_{ac}^2 d_c^6}{96\rho_m d_{inter}^4} \tag{4}$$

with v_{ac} the acoustic velocity at the plane position of the cells and d_{inter} the distance inter-cells.

The combination of these forces allows the creation of layers of cells at every pressure node. In this work we used a robust approach to obtain efficient levitation and aggregation, which consists in generating pseudo standing waves in a resonant cavity. Indeed, to maximize the emitted energy, the acoustic wave is confined between the transducer and the reflector. To obtain constructive interferences between the emitted and the reflected wave, the height H of the cavity must be equal to a multiple of the half-wavelength $H = n\frac{\lambda_{ac}}{2}$.

Estimation of the cell number in one spheroid

The cavity height and the working acoustic frequency set the number of traps. In the experiments, we have used a cavity with a 10mm height and a frequency at 2.15MHz. By considering the acoustic equations and the boundary limits, we can calculate the trap number $n_{trap} = \frac{h \times 2}{\lambda} = \frac{10}{0.344} = 29$traps. With $\lambda = \frac{c_m}{f}$ the wave number and c_m the ultrasound velocity. Here we take, $c_m = 1480$m.s^{-1} and $f = 2.15$MHz. Experimentally, we obtained a number of traps about 27 closed to the theoretical expected number, as we can see on the Fig. S 1a reconstructed from the Supplementary Video 6 in supplementary material.

The dimensions of the cavity are a 10mm height and a 5mm diameter with a volume of 196μL. If we consider a concentration of 1.5 million cells/ml, we have about 3.10^5 cells in the cavity for about 30 nodes. So we can deduce that one spheroid contains 10.10^4 cells.

Another approach consists in estimating the number of cells in the monolayer at the initial time. If we assume that just after the injection, the cells aggregate into a monolayer with a hexagonal pattern (Fig. S 1c) and we consider the MSC size about ten microns diameter, we can calculate the number of cells included inside a delimited hexagon (Fig. S 1b). The averaged radius of the cluster is about 0.55mm (Fig. 1c in the main article), so the hexagon radius is formed by 55 cells . The whole number of cells is deduced by the formula given on the Fig. S 1b. We obtain a number about 9.10^3 cells which is consistent with the previous estimation.

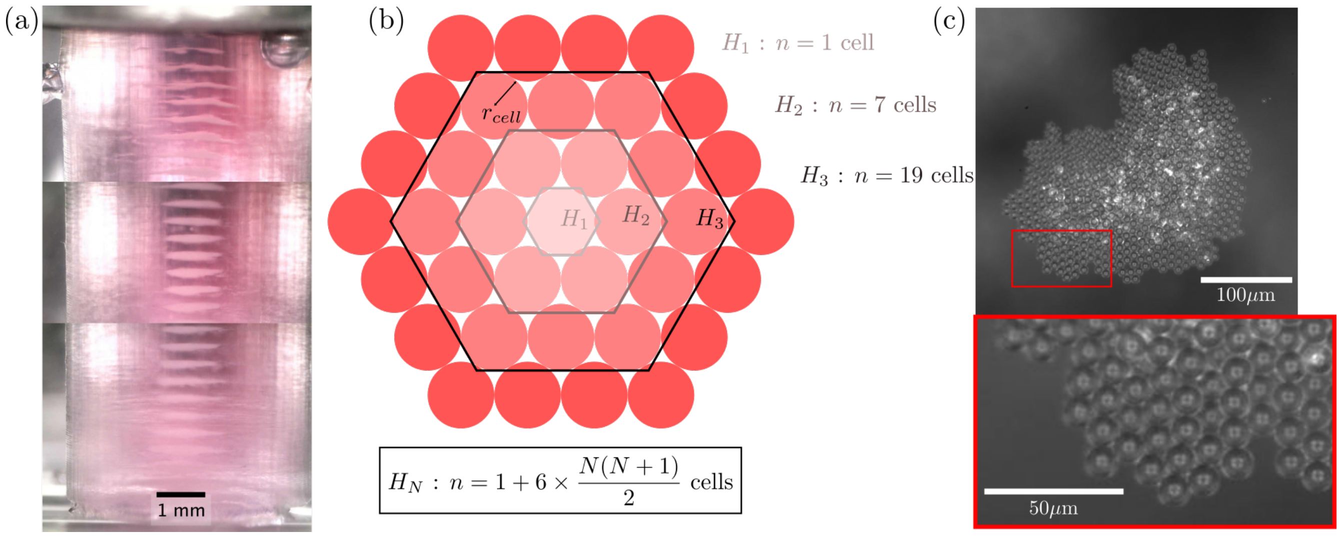

Supplementary Figure S 1. (a) Photo reconstruction of the 10mm height cavity with 29 traps. These images were taken at $t = 0$s. (b) Schema of an hexagonal grid and the formula of the cell number in a delimited hexagon. (c) Snapshot of a 10μm bead aggregate in acoustic levitation taken from the bottom view. The initial layout of the cell cluster is a monolayer with an hexagonal grid.

Results of the flow cytometry

Percentage % of positive cells	Experiment $N°1$		Experiment $N°2$		Experiment $N°3$		t-test
Markers	Levitation	2D control	Levitation	2D control	Levitation	2D control	p-value
Ig-FITC	0.43	1.96	0.376	3.152	0.78	0.46	0.1792
Ig-PE	1.9	6.89	0.296	0.715	0.52	0.61	0.4391
Ig-APC	0.42	0.49	1.047	1.9	-	-	0.6105
CD29-PE	99.81	99.73	99.22	99.61	99.47	99.5	0.5712
CD73-PE	99.97	99.99	99.93	99.79	99.8	99.96	0.8661
CD90-FITC	99.84	98.31	99.71	91.10	98.99	93.39	0.0706
CD105-PE	99.23	99.23	99.33	98.78	-	-	0.3562
CD31-FITC	0.83	2.37	0.15	0.204	-	-	0.5551
CD34-APC	0.33	0.41	8.113	7.588	-	-	0.9703

Supplementary Table S 1. Expression of the MSC surface markers

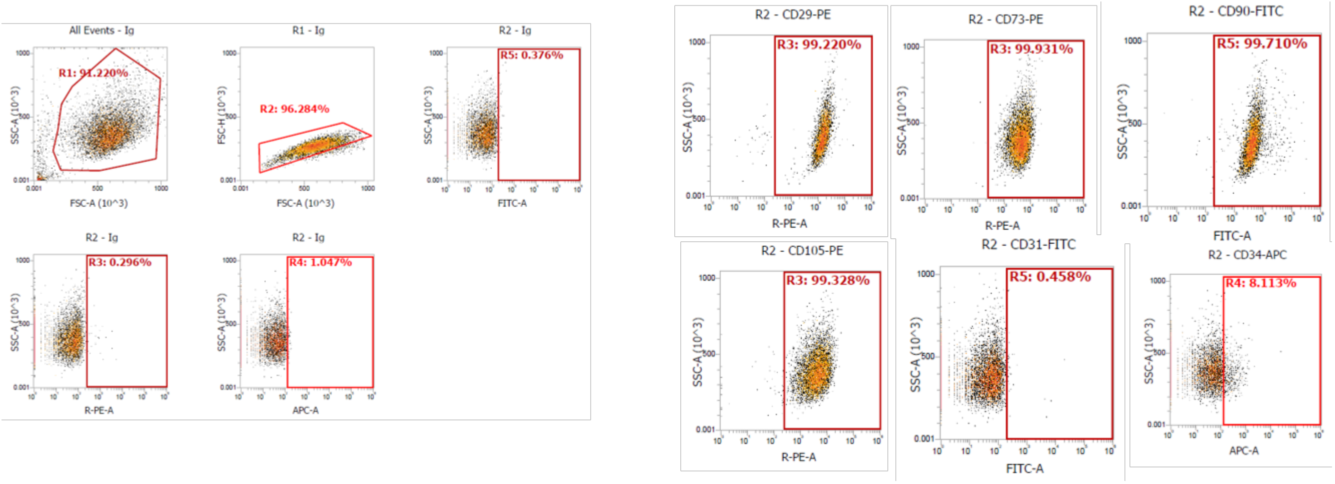

Supplementary Figure S 2. Flow cytometry evaluation of MSC under acoustic levitation

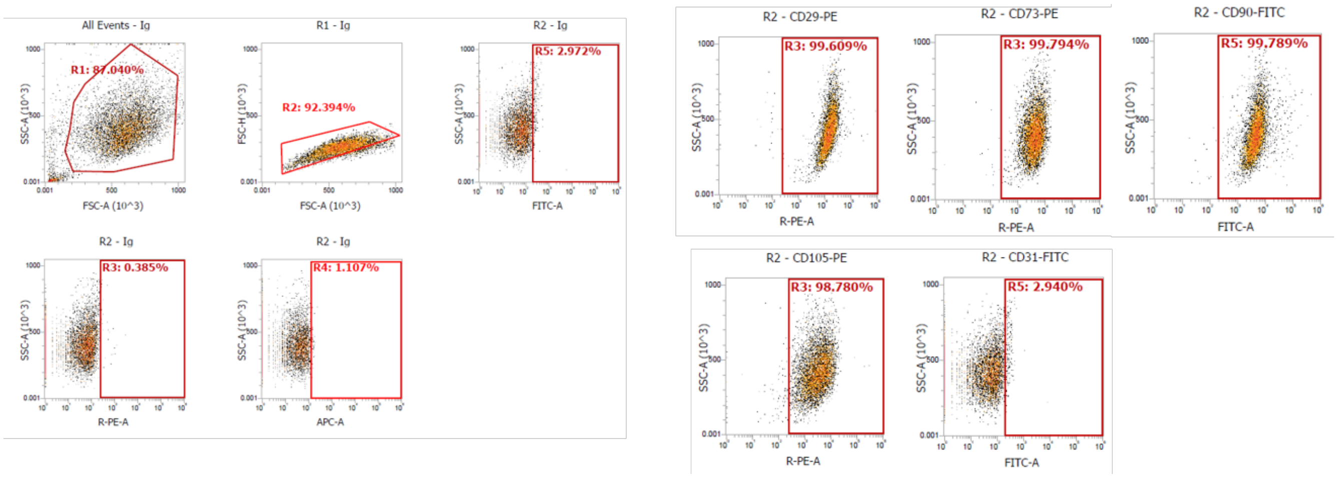

Supplementary Figure S 3. Flow cytometry evaluation of MSC in classical 2D culture

References

1. Karlsen, J. T. & Bruus, H. Forces acting on a small particle in an acoustical field in a thermoviscous fluid. *Phys. Rev. E* **92**, DOI: 10.1103/PhysRevE.92.043010 (2015).
2. Hasegawa, T. & Yosioka, K. Acoustic radiation force on fused silica spheres, and intensity determination. *The J. Acoust. Soc. Am.* **58**, 581–585, DOI: 10.1121/1.380708 (1975).
3. Gorkov, L. On the forces acting on a small particle in an acoustic field in an ideal fluid. *Sov. Physics-Doklady* **6**, 773–775 (1962).
4. Dron, O. & Aider, J.-L. Varying the agglomeration position of particles in a micro-channel using Acoustic Radiation Force beyond the resonance condition. *Ultrasonics* **53**, 1280–1287, DOI: 10.1016/j.ultras.2013.03.012 (2013).
5. Gutiérrez-Ramos, S., Hoyos, M. & Ruiz-Suárez, J. C. Induced clustering of Escherichia coli by acoustic fields. *Sci. Reports* **8**, DOI: 10.1038/s41598-018-22960-z (2018).
6. Friend, J. & Yeo, L. Y. Microscale acoustofluidics: Microfluidics driven via acoustics and ultrasonics. *Rev. Mod. Phys.* **83**, 647–704, DOI: 10.1103/RevModPhys.83.647 (2011).
